# Fibropapillomatosis and Chelonid Alphaherpesvirus 5 Infection in Kemp’s Ridley Sea Turtles (*Lepidochelys kempii*)

**DOI:** 10.3390/ani11113076

**Published:** 2021-10-28

**Authors:** Annie Page-Karjian, Liam Whitmore, Brian A. Stacy, Justin R. Perrault, Jessica A. Farrell, Donna J. Shaver, J. Shelby Walker, Hilary R. Frandsen, Elina Rantonen, Craig A. Harms, Terry M. Norton, Charles Innis, Kelsey Yetsko, David J. Duffy

**Affiliations:** 1 Harbor Branch Oceanographic Institute, Florida Atlantic University, Fort Pierce, FL 34946, USA; elirantone@gmail.com; 2 Whitney Laboratory for Marine Bioscience and Sea Turtle Hospital, University of Florida, St. Augustine, FL 32080, USA; liamwhitmore22@outlook.com (L.W.); jessicafarrell246@yahoo.com (J.A.F.); kelsey.yetsko@whitney.ufl.edu (K.Y.); duffy@whitney.ufl.edu (D.J.D.); 3 Department of Biological Sciences, University of Limerick, V94 T9PX Co. Limerick, Ireland; 4 National Oceanic & Atmospheric Administration, National Marine Fisheries Service, Gainesville, FL 32611, USA; brian.stacy@noaa.gov; 5 Loggerhead Marinelife Center, Juno Beach, FL 33408, USA; jperrault@marinelife.org; 6 Department of Biology, University of Florida, Gainesville, FL 32611, USA; 7 Division of Sea Turtle Science and Recovery, Padre Island National Seashore, Corpus Christi, TX 78480, USA; donna_shaver@nps.gov (D.J.S.); jennifer_shelby_walker@nps.gov (J.S.W.); hilary_frandsen@nps.gov (H.R.F.); 8 Center for Marine Science & Technology, North Carolina State University, Morehead City, NC 28557, USA; caharms@ncsu.edu; 9 Georgia Sea Turtle Center, Jekyll Island, GA 31527, USA; tnorton@jekyllisland.com; 10 New England Aquarium, Boston, MA 02110, USA; cinnis@neaq.org

**Keywords:** ChHV5, disease ecology, enzootic, green turtle, marine turtle, molecular diagnostics, transmission, tumor, virus, whole genome sequencing

## Abstract

**Simple Summary:**

The Kemp’s ridley sea turtle is an endangered species that is susceptible to a tumor disease called fibropapillomatosis (FP) and its associated virus, chelonid alphaherpesvirus 5 (ChHV5). The goal of our study was to describe FP in Kemp’s ridley turtles, including estimated disease prevalence and pathologyg, and case demographics and outcomes, to better understand the risk posed by FP to Kemp’s ridley population recovery. During 2006–2020, we identified 22 cases of Kemp’s ridley turtles with FP, including 12 adult turtles, a reproductively valuable age class. Molecular diagnostics were used to identify ChHV5 DNA in blood (7.8%) and tumor (91.7%) samples collected from free-ranging Kemp’s ridley turtles. Genomic sequencing was conducted to identify ChHV5 variants in tumor samples collected from Kemp’s ridley turtles with FP. Along with case data, phylogenetic analysis of resultant sequences suggests increasing, spatiotemporal spread of ChHV5 infections and FP among Kemp’s ridley turtles in coastal areas, including the Gulf of Mexico and the southwestern Atlantic Ocean, where they share habitat with green sea turtles (in which FP is enzootic). This is concerning because FP has an uncertain pathogenesis, is potentially related to anthropogenic environmental degradation, and can cause suffering and/or death in severely afflicted turtles.

**Abstract:**

Fibropapillomatosis (FP), a debilitating, infectious neoplastic disease, is rarely reported in endangered Kemp’s ridley sea turtles (*Lepidochelys kempii*). With this study, we describe FP and the associated chelonid alphaherpesvirus 5 (ChHV5) in Kemp’s ridley turtles encountered in the United States during 2006–2020. Analysis of 22 case reports of Kemp’s ridley turtles with FP revealed that while the disease was mild in most cases, 54.5% were adult turtles, a reproductively valuable age class whose survival is a priority for population recovery. Of 51 blood samples from tumor-free turtles and 12 tumor samples from turtles with FP, 7.8% and 91.7%, respectively, tested positive for ChHV5 DNA via quantitative polymerase chain reaction (qPCR). Viral genome shotgun sequencing and phylogenetic analysis of six tumor samples show that ChHV5 sequences in Kemp’s ridley turtles encountered in the Gulf of Mexico and northwestern Atlantic cluster with ChHV5 sequences identified in green (*Chelonia mydas*) and loggerhead (*Caretta caretta*) sea turtles from Hawaii, the southwestern Atlantic Ocean, and the Caribbean. Results suggest an interspecific, spatiotemporal spread of FP among Kemp’s ridley turtles in regions where the disease is enzootic. Although FP is currently uncommon in this species, it remains a health concern due to its uncertain pathogenesis and potential relationship with habitat degradation.

## 1. Introduction

The Kemp’s ridley sea turtle (*Lepidochelys kempii*) is an endangered (United States Endangered Species Act 1970)/critically endangered (International Union for Conservation of Nature) species that primarily lives in the Gulf of Mexico (GoM) and in waters off the eastern coastal United States of America (USA) [1,2,3,4]. Most Kemp’s ridley turtles nest on beaches in northeastern Mexico, although some nesting also occurs in Texas and other gulf states of the USA. Following an initial oceanic phase of their life history, juvenile and adult turtles forage in neritic habitats and prey on a variety of benthic invertebrates [5,6]. Kemp’s ridley turtles have remarkably rebounded from near-extinction due to intensive cooperative local, regional, and international conservation efforts to protect eggs and nesting females and reduce bycatch in fisheries [7]. The Kemp’s ridley is now one of the most common species admitted to sea turtle rehabilitation facilities in the USA, as it is frequently affected by cold-stunning events in the northeastern part of the country and incidentally caught by recreational fishermen in the GoM [8,9,10].

Kemp’s ridley turtles are susceptible to fibropapillomatosis (FP), a debilitating neoplastic disease of sea turtles, associated with chelonid alphaherpesvirus 5 (ChHV5) infection, and characterized by fibroepithelial tumors of the skin, eye(s), and internal organs [11,12]. Transmission of FP is known to occur horizontally through direct contact [13] and may also occur with exposure to infectious viral particles in the waters of high-use sea turtle habitats [14,15]. In green sea turtles (*Chelonia mydas*), FP is considered epizootic in many locations in the USA; it is enzootic in Florida and increasing in other locations (e.g., Texas, North Carolina) where it was previously unreported or rare [16,17,18,19]. Fibropapillomatosis is also reported with increasing frequency in sea turtle species other than green turtles [11,20,21,22,23,24,25,26,27,28]. Although imperiled sea turtle populations most affected by FP (particularly green turtles) are showing considerable signs of recovery as a result of conservation efforts, this disease remains a concern due to its wildlife and environmental health implications [29].

Historically, FP has been only rarely reported in Kemp’s ridley turtles. The first report was for a nesting Kemp’s ridley turtle encountered in 1993 at Rancho Nuevo, Mexico [20]. Records show that abnormal, FP-like skin growths were sporadically reported for Kemp’s ridley turtles nesting at Rancho Nuevo during 1985–2002 [2,30], and have been anecdotally reported from nesting beaches in Texas [2]. Unfortunately, these lesions were not assessed histologically to confirm FP. In-water surveys in Texas and Florida revealed that of more than 1000 Kemp’s ridleys captured, none had external lesions suggestive of FP [2]. In Florida, the prevalence of FP in dead or live stranded Kemp’s ridley turtles was 0.2% during 1980–2005, and 0% during 2010–2013 [31].

In recent years, however, an increasing number of Kemp’s ridley turtles have been reported with FP, including stranded turtles, nesting females, and among admissions to sea turtle rehabilitation centers [2,32] T. Norton, pers. obs. For example, in Florida during 2014–2017, seven of 746 (0.9%) stranded Kemp’s ridley turtles were reported to have FP [31]. In south Texas, including Padre Island National Seashore (the most important Kemp’s ridley nesting beach in the USA), South Padre Island, San Jose Island, and Mustang Island, out of 343 adult Kemp’s ridley turtles found stranded and 1164 observed nesting during 2007–2020, eight (0.5%) had cutaneous tumors consistent with FP (D. Shaver, pers. obs.). Although FP still appears to be relatively uncommon among Kemp’s ridley turtles, it remains one of the most important recognized infectious diseases of sea turtles and its occurrence in this species warrants further study. Therefore, with the current study, we describe FP in rehabilitating and free-ranging Kemp’s ridley sea turtles, including the life history characteristics of affected turtles and disease features encountered in recent years. We also characterize the associated virus ChHV5 to better understand its epizootiology in this species as well as its potential relationship to viral variants found in other sea turtle species.

## 2. Materials and Methods

### 2.1. Retrospective Analysis of Rehabilitation Cases and Stranding Data

To characterize FP in Kemp’s ridley turtles, we collected biological and medical data on individuals with tumors consistent with this disease. Case records for 22 turtles were shared by collaborators across the eastern United States, including sea turtle rehabilitation facilities, and nesting beach and stranding monitoring programs. Demographic and morphologic data, as well as FP tumor number, size, morphology, and histopathological (routine hematoxylin and eosin) evaluation results, were collated and analyzed for trends. Data were summarized and descriptive statistics were calculated for case data, including mean and standard deviation (SD) for standard straight carapace length (SCL) data. Fulton’s body condition index (BCI) was calculated using SCL and body mass data [33].

### 2.2. Sample Collection—Blood and Tumors

Twelve FP tumors and fifty-one blood samples taken from fifty-five Kemp’s ridley turtles were shared by collaborators for this project, including paired blood and tumor samples from four turtles, unpaired tumor samples from eight turtles, and unpaired blood samples from forty-seven turtles. Blood samples were aseptically collected from turtles’ dorsal cervical sinus (i.e., external jugular vein) into EDTA- or heparin-coated tubes using appropriately-sized needles (20–21-gauge, 1.0–1.5”) and syringes or Vacutainer (Becton Dickinson, Franklin Lakes, NJ, USA) systems. Fibropapilloma tumor sections were collected at necropsy (for *N* = 6 dead turtles) or during surgical tumor excision using a CO_2_ laser, radio surgery, or conventional surgery (scalpel), and sterile technique (for *N* = 6 live turtles). Whole blood and tumor samples were transferred to 2 mL cryogenic vials and stored at –80 °C for up to six months prior to being shipped overnight, on dry ice, to Harbor Branch Oceanographic Institute in Fort Pierce, Florida for further analysis, where they were stored at –80 °C for up to six months prior to DNA extraction.

### 2.3. DNA Extraction and qPCR for ChHV5 F-UL30 with Sequence Confirmation

Frozen tumor samples were prepared for DNA extraction by using a sterile scalpel blade to sub-section each tumor on a sterile surface and isolate a representative sample including epidermal and dermal components. Genomic DNA (gDNA) was extracted from thawed samples using the DNeasy Blood and Tissue Kit (Qiagen, Germantown, MD, USA), following manufacturer’s instructions. Extracted DNA in each sample was measured using absorbance spectrophotometry (Nanodrop) to normalize concentrations for quantitative PCR (qPCR), and ratios of absorption at 260 nm versus 280 nm were evaluated to ensure DNA purity. Extracted gDNA was stored at −80 °C for up to six months prior to qPCR analysis. A singleplex, probe-based qPCR assay was used to analyze extracted gDNA samples for the ChHV5 F-UL30 gene segment, following the assay and methodologies described in detail by Page-Karjian et al. [34]. Mean ± SD were calculated for viral copy number data of qPCR-positive samples. To further confer F-UL30 qPCR assay specificity and to confirm results, all qPCR products that tested positive were purified using the QIAquick PCR Purification Kit (Qiagen) and sequenced using Sanger sequencing (Genewiz, South Plainfield, NJ, USA) and 5 µL of 5 µM forward F-UL30 primer. Resulting sequences were compared to those in the NCBI database using BLAST software [35]. Aligned sequences with ≥95% sequence homology were considered a match and those samples were interpreted to be “positive”.

### 2.4. Whole-Genome Shotgun Sequencing of Tumor Samples

Genomic DNA from eight cutaneous tumor samples taken from seven Kemp’s ridley turtles was subjected to genome shotgun sequencing. Tumors were selected for whole genome sequencing based on sample and funding availability as well as turtle location, as samples representing Kemp’s ridley turtles from multiple different locations in the USA were prioritized. Four samples were sequenced in an untargeted manner (whole genome sequencing of host and viral genes, as previously described [14], on an Illumina NovaSeq6000 at the University of Florida’s Whitney Laboratory for Marine Biosciences and Interdisciplinary Center for Biotechnology Research Core Facilities in St. Augustine, Florida. Four samples were virally enriched (centrifugation and supernatant collection prior to DNA extraction) and sequenced on an Illumina HiSeq3000 at CD Genomics (New York, NY, USA). Sequencing data including raw reads were deposited in NCBI (https://www.ncbi.nlm.nih.gov/) (accessed on 6 April 2018) under BioProject ID: PRJNA449022 (https://www.ncbi.nlm.nih.gov/bioproject/PRJNA449022) (accessed on 6 April 2018). The resulting sequences were assessed for quality (FastQC), trimmed (Babraham Bioinformatics—Trim Galore!), and aligned to the ChHV5 reference genome (GenBank HQ878327.2) using Bowtie2 alignment software on the Galaxy EU platform [36] https://usegalaxy.eu/ (accessed on 12 September 2018). Virtual alignment was performed to map reads to ChHV5 genes, and count tables were produced to determine the number (counts) of aligned reads per gene (htseq-count, v.0.9.1). Transcripts per million (TPM) normalization was conducted manually to generate TPM graphs for all eight samples, from ChHV5 aligning reads only (Supplemental Table S3). Two samples (*Lk16*-1 and *Lk16*-2; from the same individual, moderately decomposed) had extremely low ChHV5 aligning reads and were therefore removed from phylogenetic analysis. For the remaining six samples, consensus sequences were created using aligned BAM files and the original reference ChHV5 genome FASTA sequence (GenBank HQ878327.2), with the Ococo caller program (v.0.1.2.6) from Galaxy. These six consensus sequences were then used for gene-level phylogenetic comparisons of the F-UL30 gene as well as the hypothetical protein-32 (HP32) gene, a previously identified gene with predicted immunoglobulin features [37]. HP32 was selected because the samples analyzed by shotgun sequencing had consistently higher normalized HP32 read counts than UL30 read counts.

#### 2.4.1. Viral Phylogenetics

Two phylogenetic trees were constructed using Mega X software to demonstrate the phylogenetic relationship between the six consensus sequences obtained from the Kemp’s ridley tumor samples (for F-UL30 and HP32), the ChHV5 reference genome (for F-UL30 and HP32) as well as available NCBI GenBank database-deposited ChHV5 sequences for F-UL30. Generated F-UL30 gene sequences were compared with available Atlantic and Pacific variants of this gene (Supplemental Table S4). The evolutionary history was inferred using the maximum likelihood method (https://www.megasoftware.net/) (accessed on 2 November 2018) with the Tamura-Nei model. The evolutionary distances were computed using the maximum composite likelihood method and are presented on tree branches in the number of base substitutions per site. Codon positions included were 1st + 2nd + 3rd + Noncoding. Generated F-UL30 gene sequences for phylogenetic analysis were trimmed to the same length (483 bp) and position in the genome using available sequences from the NCBI database (https://www.bioinformatics.org/sms2/range_extract_dna.html) (accessed on 22 March 2019).

#### 2.4.2. Kemp’s Ridley Mitochondrial Genome Analysis

To discern the relatedness of the ‘New England’ Kemp’s ridley turtle (*Lk*15; the northernmost reported case of FP to date) with other known turtles inhabiting the Atlantic and GoM, we used phylogenetic analysis to evaluate its full mitochondrial genome. First, the sequenced data for this turtle was aligned to the largest Kemp’s ridley mitochondrial genome available on NCBI (https://www.ncbi.nlm.nih.gov/; accession: MN136056.1) (accessed on 11 November 2020) using Galaxy (https://usegalaxy.eu/) (accessed on 11 November 2020) *Bowtie2* alignment program. All other available Kemp’s ridley mitochondrial sequences in the NCBI nucleotide database (https://www.ncbi.nlm.nih.gov/nuccore) (accessed on 13 May 2021) were downloaded in FASTA format. These sequences were then attuned to the same length and genome position as available D-loop control regions of the mitochondrial genome using a range extractor (https://www.bioinformatics.org/sms2/range_extract_dna.html) (accessed on 14 November 2020). Once obtained, all sequences were input into Clustal Omega (https://www.ebi.ac.uk/Tools/msa/clustalo/) (accessed on 18 February 2021) to observe phylogeny of the samples.

## 3. Results

### 3.1. Retrospective Case Series Analysis

**Figure 1:**
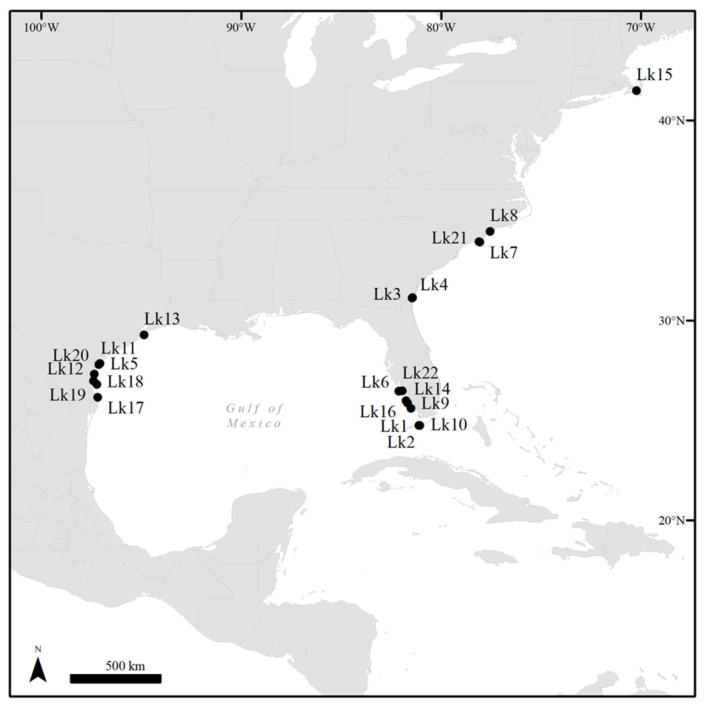
Locations of 22 cases of Kemp’s ridley sea turtles (*Lepidochelys kempii*) observed with fibropapillomatosis in the coastal United States during 2006–2020.

Overall, case data were compiled for 22 Kemp’s ridley sea turtles with FP during 2006–2020 (Table 1; Supplemental Table S1). These turtles were encountered in Texas (*N* = 8), Florida (*N* = 8), North Carolina (*N* = 3), Georgia (*N* = 2), and Massachusetts (*N* = 1), USA (Figure 1). Fourteen turtles (63.6%) were found stranded including seven (31.8%) found dead; five turtles (22.7%) were encountered while they were nesting, and three (13.6%) were incidental captures reported by fishermen. Of the 10 juvenile turtles (45.5%; SCL mean ± SD: 42.9 ± 12.6 cm, range: 26.7–59.0 cm), two were determined to be females based on visual examination of gonads at necropsy, and sex was undetermined in eight. The remaining 12 adult turtles (54.5%; SCL mean ± SD: 62.5 ± 2.0 cm, range: 60.0–66.3 cm) were females [38,39]. Subjective body condition assessments showed that two turtles (9.1%) were emaciated, four (18.2%) were thin, and 16 (72.7%) were in ‘good’ body condition. Calculated BCI ranged from 0.83 to 1.73 (mean ± SD: 1.30 ± 0.27). Eleven turtles (50.0%) were admitted to sea turtle rehabilitation facilities, including seven stranded turtles, three incidentally captured by fishers, and one nesting turtle. Of these 11 turtles, nine were admitted with tumors, and two were admitted for other reasons and developed minor FP tumors during captive care that were then surgically removed. Two of the rehabilitating turtles died, one was euthanized, and eight were released after a period of treatment and supportive care.

**Figure 2:**
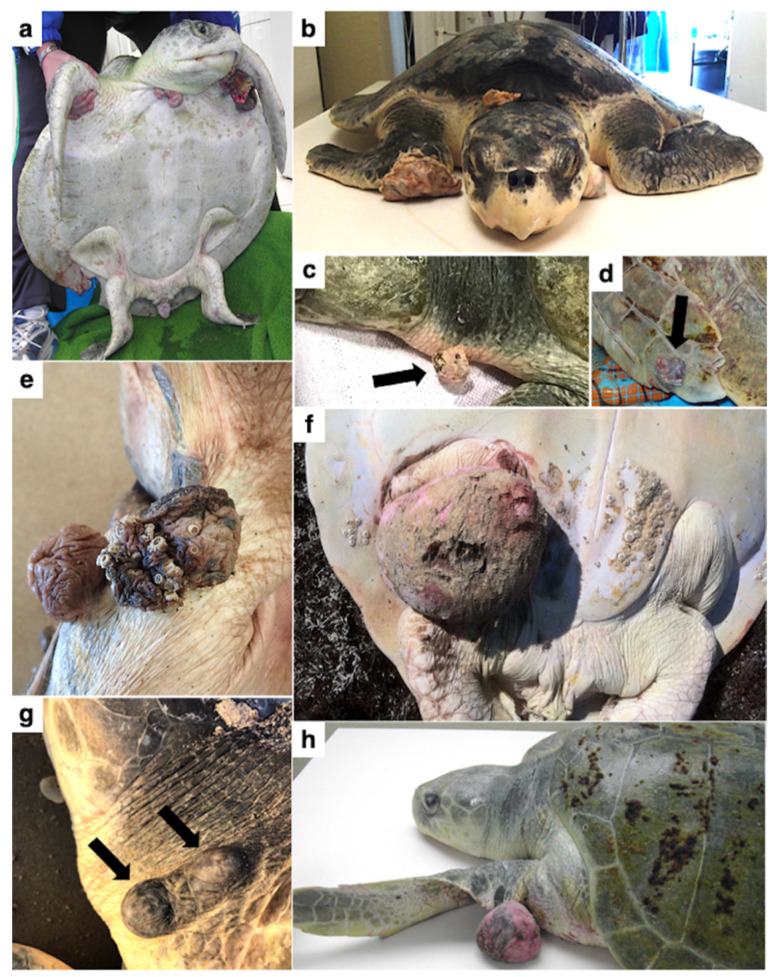
Gross morphology of fibropapillomatosis tumors in afflicted Kemp’s ridley sea turtles (*Lepidochelys kempii*). Masses can develop anywhere on a turtle’s body, and tumor morphology varies from flat plaques, to verrucous, arborizing masses (**a**–**e**), to smooth polypoid masses (**f**–**h**).

**Figure 3:**
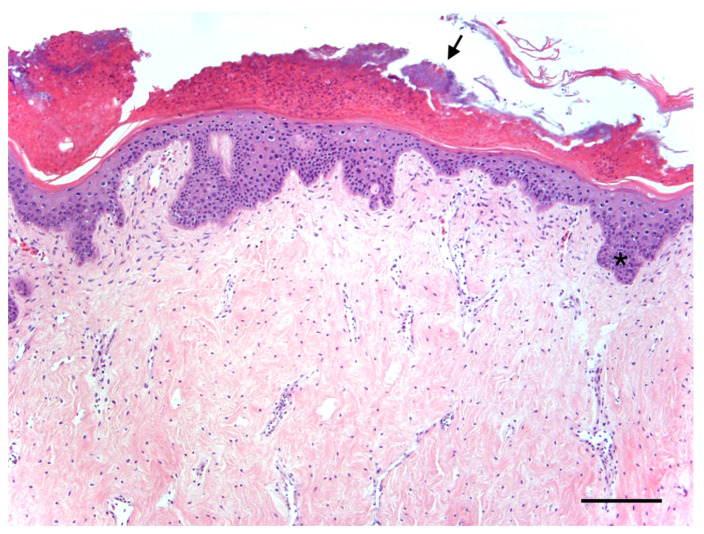
Photomicrograph of a fibropapilloma in a Kemp’s ridley sea turtle (*Lepidochelys kempii*). Histopathological features are diagnostic for this disease and include well-differentiated neoplastic epidermal and stromal components. Short epidermal pegs (*) extend into the underlying dense collagenous stroma, which includes loosely arranged streams and individually dispersed fibroblasts and supportive vessels. A crust of degenerate heterophils with accumulations of bacteria (arrow) is formed on the surface at the top of the image. Hematoxylin and eosin. Scale bar = 200 µm.

Ten turtles (45.5%) had verrucous, arborizing masses (e.g., Figure 2a–e), one (4.5%) had flat plaques, and eleven (50.0%) had smooth, pedunculated masses (e.g., Figure 2f–h). Fibropapillomatosis was diagnosed in twelve turtles (54.5%) based on pathognomonic gross features (i.e., cutaneous fibroepithelial tumors), and in nine turtles (40.9%) based on gross and histopathological examination. The most common anatomic location of tumor(s) was the neck (12/22, 54.5%), followed by the front flipper(s) (9/22, 40.9%); inguinal region(s) (6/22, 27.3%); base of front flipper(s), tail and/or tail base, and shell (each 4/22, 18.2%); internal (3/22, 13.6%); and eye(s) (2/22, 9.1%). Individual turtles had a range of 1–11 tumors per turtle, with a mean ± SD of 4 ± 4 tumors per turtle. Mean ± SD tumor diameter was 2.91 ± 0.70 cm (range: 0.25–22.0 cm). Based on the size and number of tumors, 14 turtles (63.6%) were assigned FP tumor score 1, 4 (18.2%) were tumor score 2, and 4 (18.2%) were tumor score 3 [40]. Histopathological features were as previously described for FP and included neoplastic stromal cells and epithelium supported by dense fibrovascular stroma elaborated into papillary surface projections (verrucous forms) (e.g., Figure 3). Internal tumors were composed of neoplastic stromal cells and lacked an epithelial component, except for pulmonary tumors which exhibited papillary epithelial proliferation similar to cutaneous tumors.

#### Case Report of Fibropapillomatosis in a Stranded Kemp’s Ridley Turtle in Massachusetts

**Figure 4:**
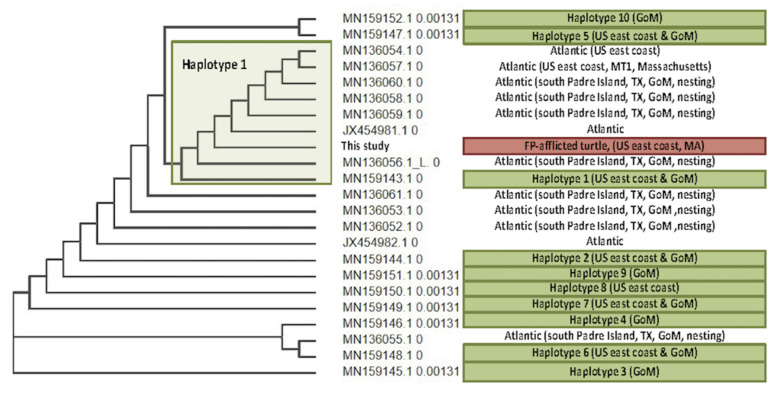
Mitochondrial DNA analysis of tissue samples collected from a Kemp’s ridley sea turtle (*Lepidochelys kempii*) that stranded in Massachusetts, USA (*Lk15*), alongside other published Kemp’s ridley mitochondrial D-loop control regions [44,45]. *Lk15* (shaded red) grouped within the haplotype 1 sub-group. Haplotype defining sequences are shaded green. Kemp’s ridley turtles along the east coast USA are 80% likely to be haplotypes 1 and 2 (predominantly haplotype 1), and 79% of haplotypes 1 and 2 originate from rookeries along the Texas coast. Combined with ChHV5 phylogenetic data (Section 3.4, Figures 6 and 7), these results suggest that *Lk15* likely originated from Texas (based on currently available data) and harbors a Florida variant of ChHV5.

In November 2019, a Kemp’s ridley turtle (*Lk15*) stranded in Brewster, Massachusetts, USA (41°46′55.146”N, 70°2′18.471”W) with a carapace fracture (possible boat strike but unconfirmed), moderate epibiota (barnacles and algae), and seven cutaneous tumors. While still a juvenile, this 56.0 cm SCL, 22.0 kg turtle was larger than those typically observed on the northeastern USA coast, which are usually in the 25–30 cm SCL size range [41]. The turtle had two cutaneous tumors on its neck (each ~3.0 × 2.0 cm), one large tumor on the right pre-femoral region (~20.0 × 22.0 cm), two on the left dorsal femoral region (~2.0 × 2.0 cm each), one on the right dorsal metacarpus (~2.0 × 2.0 cm), and one on the right shoulder (~5.0 × 2.0 cm). Grossly, the tumors were pedunculated and round with a verrucous surface. The turtle was admitted to a rehabilitation facility, and after several weeks of supportive care, the tumors were surgically excised with the turtle under general anesthesia. The excised tumors were confirmed as fibropapillomas via histopathology. Histologically, the masses were relatively typical of FP, characterized by a dense, moderately cellular stroma elaborated into thick projections and covered by well-differentiated epidermis. After tumor removal and a period of supportive care and recovery, this turtle was released into the GoM in southwestern Florida. This case is of particular interest since, to the authors’ knowledge, it represents the northernmost-occurring case of fibropapillomatosis reported to date in Kemp’s ridley turtles specifically, and in sea turtles in general [2,20,30,31,32,42]. Analysis of mitochondrial DNA from *Lk15* (mitochondrial sequence obtained from non-enriched whole genome sequencing data from this sample) along with other published Kemp’s ridley mitochondrial D-loop control regions showed that *Lk15* grouped within the haplotype 1 sub-group (Figure 4). Since previous research shows that Kemp’s ridley turtles along the east coast of the United States are 80% likely to be haplotypes 1 and 2 (predominantly haplotype 1), and that 79% of haplotypes 1 and 2 originate from rookeries along the Texas coast [43], we can infer that *Lk15* likely originated from Texas. These conclusions are based on currently available literature and may be subject to change, since Kemp’s ridley haplotype data are currently limited for regions other than Texas [43,44,45]. Phylogenetic analysis of ChHV5 UL30 and HP32 gene segments (described in detail in Section 3.4) suggests that this turtle harbored a Florida variant of ChHV5.

### 3.2. qPCR for ChHV5 DNA

Blood and tumor samples were collected from a total of 55 Kemp’s ridley turtles for molecular analysis for ChHV5 DNA. Specifically, blood samples were collected from 51 Kemp’s ridley turtles during 2015–2020, including 32 turtles in a rehabilitation facility in North Carolina, 14 free-ranging turtles hand-captured in Florida’s Big Bend region in the northeastern GoM, three nesting female turtles encountered in Texas, and two turtles in rehabilitation facilities in Texas (Supplemental Table S2). Tumor samples collected from 12 Kemp’s ridley turtles during 2015–2020 were also analyzed for ChHV5 DNA, including samples from five dead stranded turtles, four nesting turtles, and three turtles in rehabilitative care. Most blood samples (47/55, 85.5%) were collected from turtles without external tumors (Supplemental Table S2). From four turtles with FP, whole blood and tumor biopsy samples were collected concurrently. For the remaining eight turtles with FP, only tumor samples were collected (Table 1). Results of qPCR analysis of these blood and tumor samples are shown in Table 2, including mean ± SD ChHV5 DNA copy number for the 15 qPCR-positive samples (four blood samples, 11 tumor samples). Confirmatory Sanger sequencing of all qPCR-positive samples showed ≥95% identity with available ChHV5 partial genome sequences (i.e., GenBank accession number HQ878327.2) for all samples. None of the four turtles with blood samples that tested positive for ChHV5 DNA via qPCR had tumors; three were free-ranging juveniles captured at Florida’s Big Bend region, and one was a patient in a rehabilitation facility in North Carolina. None of the four turtles with FP for which both blood and tumor samples were evaluated had blood samples that tested positive for ChHV5 DNA, despite having ChHV5-positive tumor samples.

ChHV5 DNA was detected in 11/12 (91.7%) FP tumor samples using qPCR. Using a non-enriched genomics approach, ChHV5 was detected in all eight sequenced tumor samples (a subset of qPCR samples; from seven individuals), including the one sample that tested negative for ChHV5 DNA using qPCR. It is possible ChHV5 was not detected in the one qPCR-negative sample due to a low viral load, since depending on assay efficiency, sequencing-based approaches can be more sensitive and less reliant on a single gene fragment. ChHV5 alignments varied across all samples (Table 3), with a ChHV5 aligning read rate range of 2.2% to 23.2% for the four virally enriched samples, and a range of 0.01% to <0.01% for non-enriched samples. The samples with low alignment percentage have very high numbers of single nucleotide polymorphisms (SNPs), suggesting that the reference genome is not ideal. Unfortunately, this is the only currently available ChHV5 reference genome, and it was sequenced from a tumor removed from a green turtle in Hawaii with generally low alignment to Florida ChHV5 variants [46]. ChHV5 genome coverage was between 16,289× and 683× for the four virally enriched samples, and ranged from 0.07× to 33.87× for non-enriched host and viral sequenced samples (Table 3). Due to low genome coverage (0.31× and 0.07×), two samples from Marco Island, Florida were excluded from further analysis.

### 3.3. TPM Normalization

**Figure 5:**
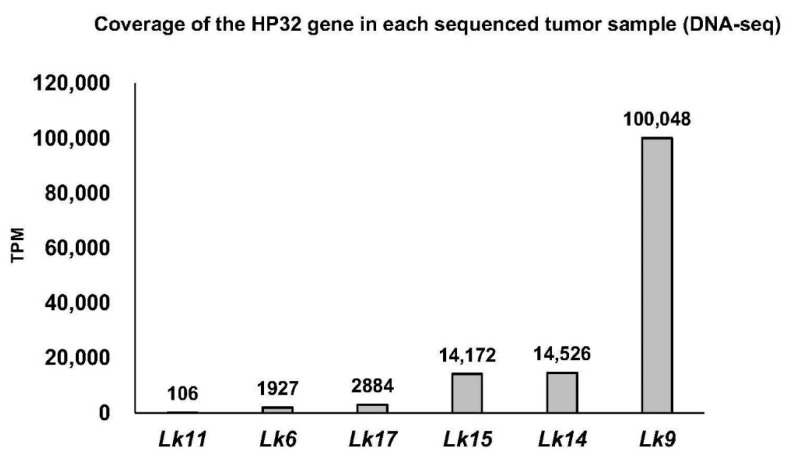
Coverage of chelonid alphaherpesvirus 5 (ChHV5) hypothetical protein-32 (HP32) gene in each of the Kemp’s ridley sea turtle (*Lepidochelys kempii*) DNA-seq samples in transcripts per kilobase million (TPM). HP32-aligning reads in these samples ranged from 3 to 31,209 reads.

The number of UL30 aligning reads were determined for each sequenced FP sample by generating count tables and calculating the number of UL30 transcripts per million (TPM) for each sample. Normalized read counts for UL30 were variable across the samples and tended to be low, with only half of the samples having UL30 TPM >100. For example, *Lk11*FPT1 and *Lk6*FPT4 have only 3 and 14 reads aligning to UL30, respectively. TPM was also calculated for another gene, HP32, resulting in relatively good TPM levels (105–100,048 reads; Figure 5). Therefore, HP32 was also selected for further phylogenetics analysis.

### 3.4. Viral Phylogenetics

**Figure 6:**
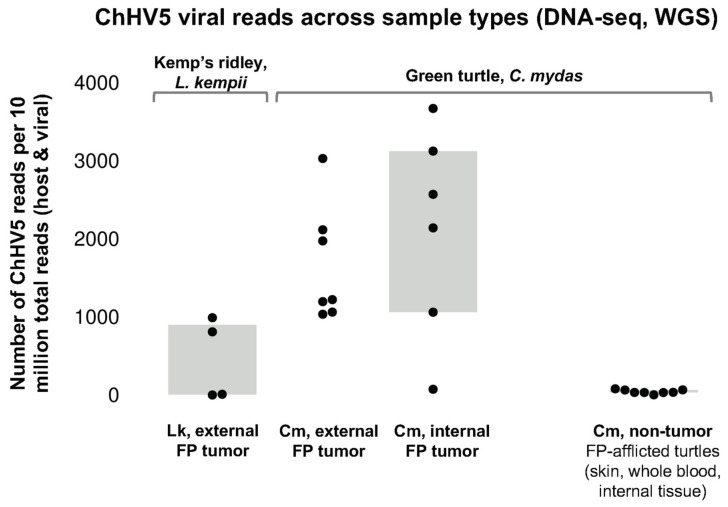
Chelonid alphaherpesvirus 5 (ChHV5) viral reads across sample types based on results of whole-genome sequencing (DNAseq). Comparing tumor ChHV5 loads (reads per ten million) shows significantly lower viral loads in samples from Kemp’s ridley sea turtles (*Lepidochelys kempii*) versus those typically observed in green sea turtles (*Chelonia mydas*; green turtle ChHV5 data adapted from Farrell et al. [14]). Abbreviations: ChHV5, chelonid alphaherpesvirus 5; Cm, *C. mydas*; FP, fibropapillomatosis; Lk, *L. kempii*; WGS, whole genome sequencing.

**Figure 7:**
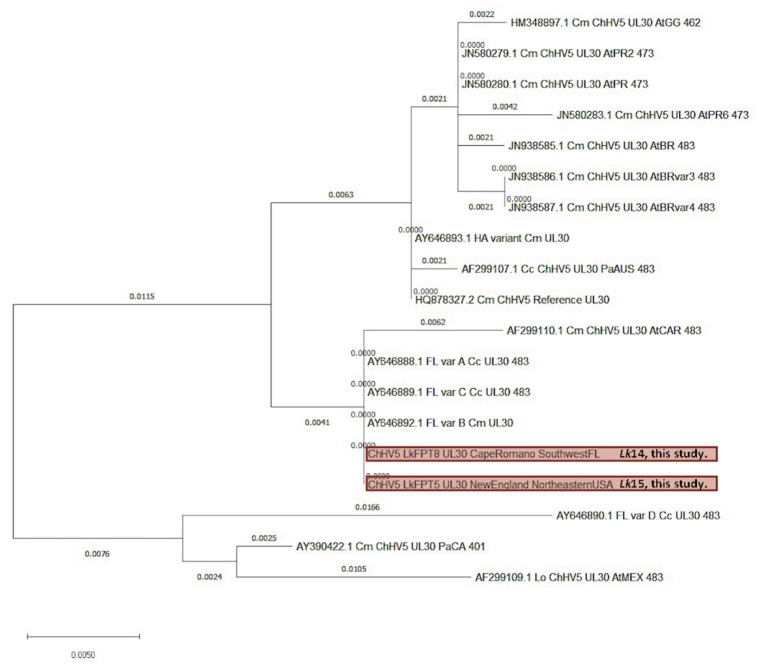
Phylogenetic analysis of partial chelonid alphaherpesvirus 5 (ChHV5) UL30 consensus genes obtained from two Kemp’s ridley sea turtle (*Lepidochelys kempii*) tumor samples along with the reference UL30 gene (GenBank accession: HQ878327.2) and other available ChHV5 UL30 genes obtained from the NCBI GenBank database, including Florida variants. Generated sequences denote turtles’ geographic locations. Generated sequences match length and position of known variants (483 bp) for direct phylogenetic comparison. The tree is drawn to scale, with branch lengths measured in the number of substitutions per site. All samples, where appropriate, have accession numbers. The two Kemp’s ridley ChHV5 gene sequences analyzed in the current study are shaded in red. Abbreviations: Cc, *Caretta caretta*; Cm, *Chelonia mydas*; Lk, *L. kempii*; Lo, *Lepidochelys olivacea*; At, Atlantic; Pa, Pacific; GG, Gulf of Guinea; PR, Puerto Rico; BR, Brazil; HA, Hawaii; AUS, Australia; CAR, Caribbean; FL, Florida; CA, California; MEX, Mexico.

For the two genes analyzed, phylogenies of most samples were in agreement, and maintained their relative phylogenetic positions throughout, based on geography. These ChHV5 reads were lower than those typically observed in green turtles (Figure 6) [14]. To note, two tumor samples from one turtle (Table 3: *Lk16*FPT6, *Lk16*FPT7) were not included in the phylogenetic analysis due to extremely low viral reads, and only two of the remaining six Kemp’s ridley sequence samples had sufficient reads for phylogenetic analysis (Table 3: *Lk15*FPT5, *Lk14*FPT8) of the ChHV5 UL30 gene. UL30 for these two remaining samples cluster closely with known Florida variants A–C, suggesting that these two turtles likely harbor one of these known variants. These samples also somewhat cluster with a Caribbean variant. Notably, these near-identical UL30 genes originate from three different sea turtle species– Kemp’s ridley, green, and loggerhead (*Caretta caretta*); Figure 7). This phylogenetic analysis further confirms previous reports that ChHV5 has regional specificity over species specificity, as similar/more closely related variants of ChHV5 have been found in sympatric species [47,48]. Florida variant D and AtMEX samples from loggerhead and olive ridley turtles (*Lepidochelys olivacea*) remain as outgroups.

**Figure 8:**
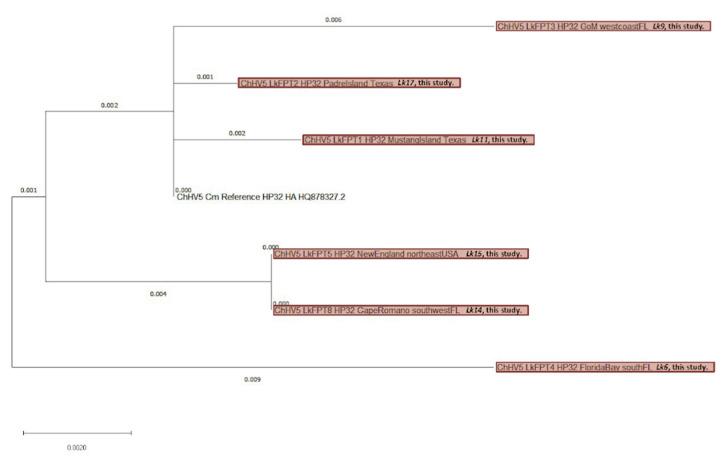
Phylogenetic analysis of chelonid alphaherpesvirus 5 (ChHV5) hypothetical protein-32 (HP32) consensus gene obtained from six Kemp’s ridley sea turtle (*Lepidochelys kempii*) tumor samples, along with the reference HP32 gene from the NCBI GenBank database (GenBank accession: HQ878327.2) sequenced from a green turtle (*Chelonia mydas*) in Hawaii. Generated sequences denote the geographic location of each turtle. Sequences match the length and position of the reference gene (1685 bp) for direct phylogenetic comparison. The tree is drawn to scale, with branch lengths measured in the number of substitutions per site. Samples generated as part of the current study are shaded red. Abbreviations: Cm, *C. mydas*; Lk, *L. kempii*; HA, Hawaii.

The TPM data showed that HP32 had the most consistently high read counts across all six sequenced tumor samples. Therefore, this gene was selected for phylogenetic comparison between all sequenced samples (including those with insufficient UL30 reads) and compared to the reference ChHV5 HP32 gene. *Lk11*FPT1, *Lk17*FPT2, and *Lk9*FPT3 all cluster closely (Figure 8), and are from individuals that were inhabiting the GoM at the time of sampling. Unexpectedly however, these samples are most closely related to the reference HP32 gene from Hawaii. *Lk15*FPT5 and *Lk14*FPT8 cluster tightly within a separate clade most closely related to Florida ChHV5 variants A–C (F-UL30 phylogeny, Figure 7). Finally, *Lk6*FPT4, which is geographically closest with *Lk11*FPT1, *Lk17*FPT2, *Lk9*FPT3, and *Lk14*FPT8, exists somewhat as an outgroup and does not exhibit clustering with any other ChHV5 HP32 gene (Figure 8).

## 4. Discussion

### 4.1. Epidemiology of FP in Kemp’s Ridley Turtles

Our study summarizes case data, including epidemiology, molecular diagnostics, and phylogenetics analysis, on Kemp’s ridley sea turtles with FP in the United States during 2006–2020. Although the current study spans a period of 14 years, nearly half (10/22, 45.5%) of these cases were reported in 2019–2020, suggesting a potential for increasing disease prevalence over time, increased reporting, or both. Of the 22 cases, 16 (72.7%) were encountered on the GoM (Texas and western Florida), and six (27.3%) were found stranded on the coasts of the western Atlantic Ocean (Georgia, North Carolina, Massachusetts), corresponding to the known range of Kemp’s ridley turtles in coastal United States waters [1,49,50]. Notably, of the 10 cases from 2019–2020, seven stranded in regions where FP is only sporadically reported (five from Texas, one from North Carolina, one from Massachusetts), including the northernmost report of FP in any sea turtle species to date. Along with data from FWC and Padre Island National Seashore that suggest increasing FP prevalence in recent years (detailed above), the data presented here suggest spatial and temporal spread of FP among Kemp’s ridley turtles [31] (D. Shaver, pers. obs.), which also coincides with the spatial and temporal spread of FP and ChHV5 among green turtles in the GoM and northward along the Atlantic seaboard of the United States. For example, although green turtle FP was only first confirmed in Texas in 2010 despite thousands of physical examinations since 1980, FP prevalence in Texas’ neritic green turtles rapidly increased from <4% in 2010 to >35% in 2018 [18,19,51]. Additionally, ChHV5 transcripts were recently examined for green turtles that stranded with FP in Texas [52], and recent studies report FP and ChHV5 in green and loggerhead turtles in Georgia and North Carolina [27,34,42]. Thus, it is plausible that increasing FP among green turtles in the GoM and the southwestern Atlantic has led to more interspecific disease transmission opportunities, resulting in increasing prevalence in Kemp’s ridley turtles in these regions as well. Phylogenetic evidence supports this hypothesis, since ChHV5 sequences found in Kemp’s ridley turtles encountered in the GoM and northwestern Atlantic cluster with ChHV5 sequences identified in green and loggerhead turtles from the southwestern Atlantic Ocean and Caribbean. This includes the one turtle that likely originated in the GoM and subsequently stranded in Massachusetts, infected with a ChHV5 variant that aligns closely to Florida ChHV5 variants A–C. Thus, it is also possible that adult female Kemp’s ridley turtles migrating between foraging sites in Florida and nesting beaches in Texas [53] may help amplify FP transmission to newly recruited, neritic green turtles in Texas.

Overall, the FP cases reported here were generally less severe than those reported in sympatric green turtles, including low numbers of mostly small tumors (1–11 tumors per turtle, 2.9 ± 1.0 cm tumor diameter, 63.6% of turtles assigned tumor score 1). FP presented as one to multiple smooth, pedunculated tumors in 50% of cases, and as verrucous-type tumors in 45.5% of cases. This is similar to descriptions of FP in loggerhead turtles, which typically present with few small tumors at very low prevalence [27], and differs from the mostly verrucous-type tumor morphology commonly observed in FP-afflicted green turtles in the eastern United States at high regional prevalence [18,54]. Species-specific differences in host-pathogen interactions, such as differing immunological responses to infection and differential pathways to cellular proliferation in tumor tissues, likely explain these disparities in disease presentation. Most (72.7%) of the Kemp’s ridley turtles with FP described here were in good body condition and did not present with co-morbidities, whereas green turtles with FP often present with emaciation, anemia, heavy epibiota, and/or co-occurring illnesses or injuries [32,55].

Additionally, most (68.1%) of the Kemp’s ridley turtles with FP were classified as adults based on SCL ≥60.0 cm [38], including five adult females encountered while nesting. This differs from what is typically seen in green turtles, which are most commonly afflicted with FP as juveniles [24]. In Florida for example, where sea turtle monitoring efforts are extensive, immature green turtles exhibit high FP prevalence (>50%) [16,56,57,58,59,60,61], whereas FP prevalence is comparatively very low (<1%) in all Kemp’s ridley turtles including juveniles [2,31]. Additionally, FP is only rarely reported among adult green turtles, which may be related to afflicted juveniles either succumbing to the disease or overcoming it with tumor regression as they approach adulthood [11,25]. In contrast, the mild FP typically observed in Kemp’s ridley turtles may persist as minor cutaneous mass(es) as turtles mature, rather than resulting in death. Alternatively, FP lesions may develop in adult turtles due to ChHV5 recrudescence due to the stress of migration and/or reproduction [61], or may arise following recent infection as Kemp’s ridley turtles share habitat and/or interact with infected juvenile green turtles in coastal areas in the northern GoM, as discussed above [62,63,64,65]. Adult turtles afflicted with FP may be concerning for Kemp’s ridley population recovery efforts, since this reproductively valuable size class contributes to future generations, and decreasing threats to adult and near-adult survivorship is a key priority for continued population recovery of this critically endangered species [2].

### 4.2. Molecular Diagnostics for ChHV5

The qPCR assay used herein was successful in amplifying ChHV5 DNA in all but one (91.7%) of the tumor samples tested. This is promising because although this qPCR assay was developed based on a consensus sequence intended to target ChHV5 UL30 gene segments in green turtle samples, it also provides reliable results when used on samples from other sea turtle species including loggerhead and Kemp’s ridley turtles [34] (current study). While qPCR is a useful diagnostic tool, whole genome sequencing allows us to appreciate base pair differences in genes of interest that may influence the sensitivity of diagnostic assays. It also allows us to recognize host species-specific sequence differences in ChHV5 and to develop phylogenetic data from which to infer viral spread. While ChHV5 loads in FP tumors are often variable (Figure 6), the wide range of ChHV5 aligning reads observed in the sequenced samples (Table 3) may arise in part due to DNA extraction methodology. Since ChHV5 is predominantly latent in FP tumors [14], a lack of lytic viral replication may account for the relatively low ChHV5 read counts obtained from some tumor samples (e.g., *Lk17*FPT2). Furthermore, enrichment was based on centrifugation and extraction from supernatant, and thus intracellular latent ChHV5 DNA may have been underrepresented.

### 4.3. ChHV5 Phylogenetics

Globally, there are four major clades of ChHV5: eastern Pacific, midwestern Pacific, western Atlantic/eastern Caribbean, and Atlantic [48]. To date, however, no local-scale phylogenetic analysis of ChHV5 has been conducted for the western Atlantic clade, particularly in the GoM. One previous study discovered six distinct local variants of ChHV5 in northeastern Australia [66], similar to the four variants identified in Florida (A–D) [47]. In the present study, phylogenetic analysis was firstly based on the F-UL30 gene of ChHV5, a commonly used gene for herpesvirus phylogenetics as it is highly conserved. This gene has accrued small genetic changes (SNPs) attributed to ongoing positive selection, thus allowing for regional phylogenetic classification of ChHV5 [37]. We generated novel consensus sequences from six tumor samples to investigate ChHV5 in Kemp’s ridley turtles throughout the western Atlantic using F-UL30 gene sequences and the readily sequenced HP32 gene. We found that for the F-UL30 genes generated, *Lk14*FPT8 and *Lk15*FPT5 (from New England and southwest Florida) had the same or a very closely related variant to known Florida variants A–C [47]. This shows that closely related variants A–C can be confidently identified in species other than green turtles, and suggests that interspecies transmission may contribute to disease distribution. Phylogenetic analysis based on the HP32 gene revealed a close relationship between ChHV5 sequences in three samples from the GoM and sequences from the Pacific (Hawaiian green turtle reference genome; Figure 8). Since ChHV5 is not well-studied in Kemp’s ridley turtles, more research, including whole genome phylogenomics and larger sample sizes, is warranted to further understand the phylogeography of ChHV5.

## 5. Conclusions

The evidence presented here suggests that, while currently uncommon, fibropapillomatosis may be increasing in endangered Kemp’s ridley sea turtles. This is concerning since the disease has an uncertain pathogenesis, is potentially related to anthropogenic environmental degradation, and can cause suffering and/or death in severely afflicted turtles. Phylogenetic analysis of ChHV5 sequences isolated from Kemp’s ridley turtles with fibropapillomatosis suggest interspecific, spatial, and temporal spread of this disease among Kemp’s ridley turtles in regions where the disease is enzootic, including the Gulf of Mexico and the southeastern Atlantic coast of the United States.

## Figures and Tables

**Table 1:** Summary case data for Kemp’s ridley sea turtles (*Lepidochelys kempii*) with fibropapillomatosis. * denotes tumor samples analyzed using whole genome shotgun sequencing.

Turtle ID	Year	Age Class	Sex	How Encountered	Location	FP Tumor Score	# Tumors	Tumor Location(s)
*Lk1*	2006	Juvenile	Undetermined	Stranded, rehabilitation	Florida	3	8	Neck, LF and LH flippers, carapace, plastron, tail
*Lk2*	2006	Juvenile	Undetermined	Stranded, rehabilitation	Florida	1	1	LF flipper
*Lk3*	2011	Juvenile	Undetermined	Stranded, rehabilitation	Georgia	1	8	L/R inguinal regions, tail base
*Lk4*	2011	Juvenile	Undetermined	Incidental capture, rehabilitation	Georgia	1	2	LF and RF flippers
*Lk5*	2012	Adult	Female	Nesting, rehabilitation	Texas	2	1	R inguinal region
*Lk6* ^a,c^	2014	Adult	Female	Stranded dead	Florida	3	9	*Neck*, carapace, R inguinal region, tail, skeletal muscle, L/R lungs
*Lk7*	2014	Juvenile	Undetermined	Stranded, rehabilitation	North Carolina	1	1	Tail
*Lk8*	2014	Juvenile	Undetermined	Incidental capture, rehabilitation	North Carolina	1	1	LF flipper
*Lk9* ^a,c^	2015	Juvenile	Female	Stranded dead	Florida	2	8	*Neck*, LF and RF flippers, carapace
*Lk10*	2015	Adult	Female	Stranded, rehabilitation	Florida	3	11	R eye, neck, LF and RF flippers, R inguinal region, L/R lungs
*Lk11* ^a,c^	2017	Juvenile	Undetermined	Stranded dead	Texas	1	1	*L inguinal region*
*Lk12*	2017	Adult	Female	Stranded dead	Texas	2	10	Neck, LF and RF flipper bases
*Lk13* ^a^	2019	Adult	Female	Incidental capture, rehabilitation	Texas	1	2	Neck, RF flipper base
*Lk14* ^a,c^	2019	Adult	Female	Stranded dead	Florida	1	2	*Neck*
*Lk15* ^a,c^	2019	Juvenile	Undetermined	Stranded, rehabilitation	Massachusetts	2	7	*Neck*, RF flipper, RF and LH flipper bases, R inguinal region
*Lk16* ^a,c^	2019	Juvenile	Female	Stranded dead	Florida	1	2	*Neck, LF flipper*
*Lk17* ^a,b,c^	2019	Adult	Female	Nesting	Texas	1	1	*LF flipper* *
*Lk18* ^a^	2020	Adult	Female	Nesting	Texas	1	2	Neck
*Lk19* ^a,b^	2020	Adult	Female	Nesting	Texas	1	1	Neck
*Lk20* ^a,b^	2020	Adult	Female	Nesting	Texas	1	2	L eye, neck
*Lk21* ^a^	2020	Adult	Female	Stranded, rehabilitation	North Carolina	1	2	RF flipper base
*Lk22* ^a^	2020	Adult	Female	Stranded dead	Florida	3	4	L shoulder, L/R lungs

^a^ tumor collected; ^b^ tumor and blood collected; ^c^ whole genome sequencing performed. *Italicized* tumor locations represent which tumors were sampled for molecular analysis. Abbreviations: FP, fibropapillomatosis; L, left; R, right; F, front; H, hind.

**Table 2:** Summary of qPCR data on ChHV5 DNA copy numbers in whole blood and fibropapilloma samples collected from Kemp’s ridley sea turtles (*Lepidochelys kempii*). Gene target of the qPCR assay is ChHV5 UL30 (i.e., GenBank HQ878327.2).

Sample Type	*N*	Positive for ChHV5 DNA *N* (Prevalence)	Mean (± SD) ChHV5 DNA Copy Number (Range)
Whole blood	51	4 (7.8%)	649 ± 516 (210–2393)
FP tumors	12	11 (91.7%)	6,809,552 ± 5,446,113 (50–17,261,001)

Abbreviations: ChHV5, chelonid alphaherpesvirus 5; qPCR, quantitative polymerase chain reaction; SD, standard deviation.

**Table 3:** Tumor samples from six Kemp’s ridley sea turtles (*Lepidochelys kempii*) with unique ID code, location of turtle used for sampling, viral enrichment status, total reads for each sequenced sample, percentage alignment to the whole ChHV5 genome, total aligning reads to the ChHV5 genome, number of reads aligning to ChHV5 per 10 million total reads, ChHV5 genome coverage, and number of single nucleotide polymorphisms (SNPs). Tumor samples six and seven are from the same individual (*Lk16*).

Sample	Location	Viral Enrichment (VE)	Total Reads	% ChHV5 Alignment	Total ChHV5 Aligning Reads	ChHV5 Reads per 10 Million Total Reads	ChHV5 Genome Coverage (x)	Number of SNPs	Sample Acquisition
*Lk11* **FP Tumor 1**	Mustang Island, TX	VE	34,533,962	14.61	4,222,944	1,461,284	9580.69	106	Necropsy
*Lk17* **FP Tumor 2**	N. Padre Island, TX	VE	14,641,416	2.20	301,155	219,649	683.24	88	Nesting
*Lk9* **FP Tumor 3**	Marco Island, SW FL	VE	58,980,497	13.10	6,527,585	1,309,565	14,809.28	438	Necropsy
*Lk6* **FP Tumor 4**	Florida Bay, SW FL	VE	36,964,405	23.20	7,180,057	2,320,447	16,289.56	341	Necropsy
*Lk15* **FP Tumor 5**	Brewster, MA	VE	111,730,494	0.01	9062	811	20.56	2223	Surgery
*Lk16-1* **FP Tumor 6**	Sanibel, SW FL	Non-VE	129,584,639	<0.01	140	11	0.31	N/A	Necropsy
*Lk16-2* **FP Tumor 7**	Sanibel, SW FL	Non-VE	197,167,284	<0.01	30	2	0	N/A	Necropsy
*Lk14* **FP Tumor 8**	Cape Romano, SW FL	Non-VE	150,443,070	0.01	14,929	992	33.87	2284	Necropsy

Abbreviations: ChHV5, chelonid alphaherpesvirus 5; FL, Florida; FP, fibropapillomatosis; *Lk*, *L. kempii*; MA, Massachusetts; SNPs, single nucleotide polymorphisms; SW, southwest; TX, Texas; VE, viral enrichment.

## Data Availability

Raw case data are openly available in FigShare at doi:10.6084/m9.figshare.15185853.

## Supplementary Materials

The following are available online at https://www.mdpi.com/article/10.3390/ani11113076/s1. Supplemental Table S1: Case data for 22 Kemp’s ridley (*Lepidochelys kempii*) sea turtles with fibropapillomatosis encountered in the USA during 2006–2020; Supplemental Table S2: Blood samples were collected from 55 Kemp’s ridley (*Lepidochelys kempii*) sea turtles encountered in the United States during 2006–2020. Genomic DNA extracted from whole blood samples was tested for chelonid alphaherpesvirus 5 (ChHV5) DNA using quantitative polymerase chain reaction (qPCR), after Page-Karjian et al. (2015) [34]; Supplemental Table S3: Number of aligning reads and transcripts per million (TPM) values for every chelonid alphaherpesvirus 5 (ChHV5) gene for each sample used in this study; Supplemental Table S4: GenBank accession numbers, phylogenetic tree chelonid alphaherpesvirus 5 (ChHV5) gene name and identifier, and sea turtle species, followed by location of where turtle was sampled for use in the current study. Abbreviations: Cc, *Caretta caretta*; Cm, *Chelonia mydas*; Lk, *Lepidochelys kempii*; Lo, *Lepidochelys olivacea*; At, Atlantic; Pa, Pacific; GG, Gulf of Guinea; PR, Puerto Rico; BR, Brazil; HA, Hawaii; AUS, Australia; CAR, Caribbean; FL, Florida; CA, California; MEX, Mexico.
